# Downhill Running-Based Overtraining Protocol Improves Hepatic Insulin Signaling Pathway without Concomitant Decrease of Inflammatory Proteins

**DOI:** 10.1371/journal.pone.0140020

**Published:** 2015-10-07

**Authors:** Alisson L. da Rocha, Bruno C. Pereira, José R. Pauli, Dennys E. Cintra, Claudio T. de Souza, Eduardo R. Ropelle, Adelino S. R. da Silva

**Affiliations:** 1 Postgraduate Program in Rehabilitation and Functional Performance, RibeirãoPreto Medical School, USP, RibeirãoPreto, São Paulo, Brazil; 2 Sport Sciences Course, Faculty of Applied Sciences, State University of Campinas, Limeira, São Paulo, Brazil; 3 Exercise Biochemistry and Physiology Laboratory Postgraduate Program in Health Sciences, Health Sciences Unit, University of Far Southern Santa Catarina, Criciúma, Santa Catarina, Brazil; 4 School of Physical Education and Sport of RibeirãoPreto, University of São Paulo, RibeirãoPreto, São Paulo, Brazil; Univeristy of California Riverside, UNITED STATES

## Abstract

The purpose of this study was to verify the effects of overtraining (OT) on insulin, inflammatory and gluconeogenesis signaling pathways in the livers of mice. Rodents were divided into control (CT), overtrained by downhill running (OTR/down), overtrained by uphill running (OTR/up) and overtrained by running without inclination (OTR). Rotarod, incremental load, exhaustive and grip force tests were used to evaluate performance. Thirty-six hours after a grip force test, the livers were extracted for subsequent protein analyses. The phosphorylation of insulin receptor beta (pIRbeta), glycogen synthase kinase 3 beta (pGSK3beta) and forkhead box O1 (pFoxo1) increased in OTR/down versus CT. pGSK3beta was higher in OTR/up versus CT, and pFoxo1 was higher in OTR/up and OTR versus CT. Phosphorylation of protein kinase B (pAkt) and insulin receptor substrate 1 (pIRS–1) were higher in OTR/up versus CT and OTR/down. The phosphorylation of IκB kinase alpha and beta (pIKKalpha/beta) was higher in all OT protocols versus CT, and the phosphorylation of stress-activated protein kinases/Jun amino-terminal kinases (pSAPK-JNK) was higher in OTR/down versus CT. Protein levels of peroxisome proliferator-activated receptor-gamma coactivator 1alpha (PGC-1alpha) and hepatocyte nuclear factor 4alpha (HNF-4alpha) were higher in OTR versus CT. In summary, OTR/down improved the major proteins of insulin signaling pathway but up-regulated TRB3, an Akt inhibitor, and its association with Akt.

## Introduction

High intensity and volume exercise sessions are generally used during training programs to disrupt cellular homeostasis, induce overcompensation and improve physical performance. However, the optimal training adaptations require adequate periods of recovery because high-load exercise sessions may lead to temporary performance decreases and acute fatigue [1]. The imbalance between training demands and sufficient recovery may lead to functional overreaching (FOR), nonfunctional overreaching (NFOR) or overtraining syndrome (OTS) [2]. According to Meussen and coworkers [2], FOR occurs after an increased training load and is characterized by a short-term performance decrement without severe psychological symptoms or other lasting negative symptoms. In addition, after days of recovery, FOR eventually leads to an improvement in performance.

Athletes who do not respect this recovery period may experience NFOR, a performance decrement that may be reversed after weeks or months of recovery and may be related to psychological and hormonal disturbances. In OTS, however, the performance decrement can last for months to years, and it is difficult to distinguish between NFOR and OTS, as both may share the same signs and symptoms [2]. Pereira et al. [3] verified that mice subjected to an eight-week overtraining (OT) protocol based on eccentric exercise (EE) presented NFOR and increased the protein levels of interleukin–6 (IL–6), myostatin and tumor necrosis factor-alpha (TNF-alpha) in skeletal muscle samples as well as serum levels of IL–6 [4].

Recently, Pereira et al. [5] showed this same OT protocol impaired the skeletal muscle insulin signaling pathway (i.e. decreased the phosphorylation of insulin receptor beta/IRbeta and of the protein kinase B/Akt, and increased the phosphorylation of insulin receptor substrate 1/IRS–1, Ser307) with concomitant increases of theIκB kinase alpha and beta (IKKalpha/beta) and of the stress-activated protein kinases/Jun amino-terminal kinases (SAPK-JNK) phosphorylations, as well as of the suppressor of cytokine signaling 3 (SOCS3) protein levels. However, the authors did not observe significant differences during the insulin tolerance test (ITT), likely because the liver had assumed a more important function in maintaining glucose homeostasis in the overtrained group [5].

The liver plays a pivotal role in the maintenance of glycemic homeostasis by balancing the uptake and storage of glucose via glycogenesis and the release of glucose via glycogenolysis and gluconeogenesis [6]. In an elegant study, Zisman et al. [7] showed that glucose was partially shunted to the liver in mice with skeletal muscle disruption of glucose transporter 4 (GLUT4). In accordance with this, Kotani et al. [8] observed that the liver assumes a much greater function for glucose homeostasis in mice lacking GLUT4 in both adipose tissue and skeletal muscle, reinforcing the hypothesis by Pereira et al. [5] that the livers of overtrained mice assume a major role in glucose homeostasis. It is known that chronic exercise improves hepatic insulin signaling and decreases hepatic inflammation in obesity and type 2 diabetes mellitus [9, 10]. In addition, by enhancing hepatic gluconeogenesis, trained rodents are able to avoid exercise-induced hypoglycemia [11], a possible symptom of OTS [12]. However, the effects of OT on hepatic insulin signaling, inflammation and gluconeogenesis remain unknown.

Thus, the first aim of the present investigation was to verify the effects of the EE sessions-induced OT protocol [3] on classical proteins related to insulin signaling (IRbeta, IRS–1/Ser307,Akt, glycogen synthase kinase 3 beta/GSK3beta, forkhead box O1/Foxo1), inflammation (IL–6, IL–10, IL–15, TNF-alpha, SOCS3, IKKalpha/beta, and SAPK-JNK) and gluconeogenesis (peroxisome proliferator-activated receptor-gamma coactivator 1alpha/PGC-1alpha, hepatocyte nuclear factor 4alpha/HNF-4alpha, phosphoenolpyruvate-carboxykinase/PEPCK, and glucose-6-phosphatase/G6Pase) in the livers of C57BL/6 mice. Our hypothesis is that chronic EE does not impair liver insulin signaling. Because EE is characterized by singular features [13] and NFOR can be induced without the predominance of EE sessions [14], our second aim was to verify whether the previously mentioned proteins respond to Pereira´s protocol [3] similarly to other two OT protocols with same intensity and volume, but performed in uphill and without inclination [15].

## Materials and Methods

### Experimental animals

Male C57BL/6 mice from the Central Animal Facility of the Ribeirão Preto campus of the University of Sao Paulo (USP) were maintained in individual cages with controlled temperature (22±2°C) on a 12:12-h light-dark inverted cycle (light: 6 PM to 6 AM, dark: 6 AM to 6 PM) with food (Purina chow) and water *ad libitum*. The experimental procedures were performed in accordance with the Brazilian College of Animal Experimentation (http://www.cobea.org.br) and were approved by the Ethics Committee of the University of Sao Paulo(ID 14.1.873.53.0). Eight-week-old C57BL/6 mice were divided into four groups: control (CT; sedentary mice; n = 18), overtrained by downhill running (OTR/down; performed the OT protocol based on downhill running; n = 18), overtrained by uphill running (OTR/up; performed the OT protocol based on uphill running; n = 18) and overtrained by running without inclination (OTR; performed the OT protocol based on running without inclination; n = 18). The CT, OTR/down,OTR/upand OTRmice were manipulated and/or trained in a dark room between 6 to 8 AM [3].

### Incremental load test (ILT)

As previously described [3–5], mice were first adapted to treadmill running (INSIGHT®, Ribeirão Preto, São Paulo, Brazil) for fivedays, for 10min.day^−1^ at 3m.min^−1^. Then, rodents performed the ILT with an initial intensity of 6m.min^−1^ at 0% with increasing increments of 3m.min^−1^ every 3min until exhaustion, as defined by the time at which the mice touched the end of treadmill five times in 1min. Mice were encouraged using physical prodding. If mice became exhausted without completing the stage, the exhaustion velocity (EV; m.min^−1^) was corrected according to Kuipers et al. [16]: EV = V + (n/b).a, where *V* is the velocity (m.min^−1^) of the last completed stage, *n* is the duration (min) maintained in the incomplete stage, *b* is the duration (min) of the stage, and *a* is the test increment (m.min^−1^). The EV of mice was used to determine the intensity of the OT protocols.

### Overtraining protocols based on downhill running, uphill running, and running without inclination

The eight-week OT protocols based on downhill running, uphill running and running without inclination were performed as previously described [15], and each experimental week consisted of five days of training followed by two days of recovery. During the first four weeks of the OT protocols (i.e. first stage), the intensity was maintained at 60% of EV and the volume was gradually increased to 60min per day in the fourth week. In this first stage, rodents ran at a grade of 0%. In the fifth week of the OT protocols, the intensity and volume were maintained, but the rodents ran at a grade of -14% (OTR/down), 14% (OTR/up) or 0% (OTR).

These running grades were maintained until the end of the OT protocols. In the sixth week of the OT protocols, the intensity was increased to 70% of EV. In the seventh week of the OT protocols, the intensity and volume were increased to 75% of EV and 75min, respectively. In the eighth week of the OT protocols, the number of daily sessions was doubled. The rest interval between daily sessions during the eighth week was 4h. From the fifth week of the OT protocols, the training volume (min) performed by each experimental group was recorded daily. Considering that the life expectancy for humans is approximately 80 years and for C57BL/6 mice is approximately two years [17], eight weeks of overtraining and two weeks of recovery in mice are equivalent to 320 and 80 weeks in humans, respectively.

### Performance evaluations

The performance evaluations of the experimental groups were performed on week 0 and 48h after the last sessions of the OT protocols at the end of weeks 4 and 8. This consisted of the rotarod test [18], the ILT [3–5, 15], the exhaustive test [3–5, 15] and the grip force test [19]. On week 0, the experimental groups performed the ILT without inclination. However, at the end of weeks 4 and 8, the CT and OTR performed the ILT without inclination, the OTR/down performed the ILT in downhill running, and the OTR/up performed the ILT in uphill running. For the ILT and exhaustive test at the end of week 8, blood samples were taken from the tails of mice using 25-μL heparinized capillary tubes before the evaluations and at 0, 3, 5 and 7 min after the evaluations. Blood lactate concentrations (mM) were assayed using a lactate analyzer (YSI 1500 Sport, Yellow Spring Instruments, OH, USA).

#### Rotarod test

Motor coordination and balance were evaluated using an accelerating single-station rotarod treadmill (INSIGHT®, Ribeirão Preto, São Paulo, Brazil). Mice were placed one at a time on the rotarod treadmill with an initial intensity of 1 rpm and a final intensity of 40 rpm that was reached 300seconds later. Mice performed three consecutive trials and the mean time that each rodent was able to stay on the top of the rotarod treadmill was recorded [18]. Four hours after the rotarod test, mice performed the ILT [3–5, 15].

#### Exhaustive test

Twenty-four hours after the ILT, mice ran at 36m.min^−1^ with 8% treadmill grade until exhaustion which was determined when mice touched the end of treadmill five times in 1 min as previously described [3–5, 15]. Mice were encouraged using physical prodding. This value was recorded as the time to exhaustion (s).

#### Grip force test

Four hours after the exhaustive test, mice performed the grip force test. The researcher gently held each mouse by the tail and allowed it to grasp the horizontally positioned metal bar of the Grip Strength System (Avs Projetos®, São Carlos, São Paulo, Brazil) with the hindpaws. Each mouse performed three trials for adaptation and three trials for force measurement. The highest force value that was applied to the metal bar was recorded as the peak tension (N) and was used as a performance parameter [19].

### Metabolic parameters, glucose tolerance test (GTT), insulin tolerance test (ITT) and pyruvate tolerance test (PTT)

The body weight and food intake of the experimental groups were recorded daily. Food intake was determined by subtracting by the final food weight from the initial food weight, from each day, as previously described [15]. At the end of week 8, 24h after the last sessions of OT protocols, mice were injected intraperitoneally with glucose (2g.kg^−1^) after an overnight fast (12h). Blood samples from tails were collected at 0, 15, 30, 60 and 120min to measure blood glucose concentrations using a glucometer (Accu-chek; Roche Diagnostic Corp., Indianapolis, IN) [20]. For the ITT, fed mice were injected intraperitoneally with human recombinant insulin (1.5U.kg^−1^, Eli Lilly, Indianapolis, IN). Blood samples were collected at 0, 5, 10, 15, 20, 25 and 30min to measure blood glucose concentrations using the previously mentioned glucometer. The PTT was performed to estimate gluconeogenesis [21]. After 16h of fasting, mice were injected intraperitoneally with pyruvate (2g.kg^−1^, Sigma®-Aldrich, St Louis, MO) dissolved in saline. Blood samples were collected at 0, 30, 60, 90 and 120min to measure blood glucose concentrations using the previously mentioned glucometer. For the GTT, ITT and PTT, areas under the curves (AUC) were calculated using the trapezoidal principle [5].

### Liver extraction, immunoprecipitation and immunoblotting

Mice were anaesthetized 36h after the grip force test (i.e. at the end of week 8). After an overnight fast (12h), rodents were anaesthetized with an intraperitoneal (i.p.) injection of 2-2-2 tribromoethanol 2.5% (10–20μL.g^−1^). As soon as the effect of anaesthesia was confirmed by the loss of pedal reflexes, the abdominal cavity was opened, the portal vein was exposed and saline with and without human recombinant insulin (10 U.kg^−1^, Eli Lilly, Indianapolis, IN) was injected. At 30s after saline or saline with human recombinant insulin injection [22], each mouse liver was removed, minced coarsely and homogenized in extraction buffer (1% Triton X–100, 100mMTris, pH 7.4, containing 100mM sodium pyrophosphate, 100mM sodium fluoride, 10mM EDTA, 10mM sodium vanadate, 2mM PMSF and 0.1 mg.mL^−1^aprotinin) at 4°C with a Polytron PTA 20S generator (Brinkmann Instruments model PT 10/35), operated at maximum speed for 30s. The extracts were centrifuged (9900g) for 40min at 4°C to remove insoluble material, and the supernatants were used for protein quantification using the Bradford method as previously described [5].

Equal amounts of protein were used for immunoprecipitation with 10μL of the following antibodies: insulin IRbeta (SC20739) from Santa Cruz Biotechnology (Santa Cruz, CA, USA) and Akt (CELL9272S) from Cell Signaling Technology (Beverly, MA, USA). The immunocomplex was precipitated with protein A-Sepharose 6 MB (Pharmacia; Uppsala, Sweden) and washed three times with 50mM Tris (pH 7.4) containing 2mM sodium vanadate and 0.1% Triton X–100. Proteins were denatured by boiling in Laemmli sample buffer containing 100mM DTT, run on SDS-PAGE gel and transferred to nitrocellulose membranes (GE Healthcare, Hybond ECL, RPN303D). The transfer efficiency to nitrocellulose membranes was verified by brief staining of the blots with Ponceau red stain. These membranes were then blocked with Tris-buffered saline (TBS) containing 5% BSA and 0.1% tween–20 for 1 h at 4°C.

Antibodies used for immunoblotting overnight at 4°C were phospho-tyrosine (SC8954S), IRbeta (SC20739), IRS–1 (SC560), phospho-IRS–1 (Ser307; SC33956), GSK3beta (SC9166), tribbles-like protein 3 (TRB3; SC365842), S6K1 (SC74459), phospho-S6K1 (Thr389; SC11759), IL–10 (SC52561), IL–15 (SC7889), SOCS3 (SC9023), IKKbeta (SC34674), phospho-IKKalpha/beta (Ser180/181; SC23470R), PGC-1alfa (SC13067), HNF-4alfa (SC6556), PEPCK (SC271204) and beta-Actin (SC69879) from Santa Cruz Biotechnology (Santa Cruz, CA, USA); Akt (CELL9272S), phospho-Akt (Ser473; CELL4058S), phospho-GSK3beta (Ser9; CELL5558S), phospho-Foxo1 (Ser256; CELL9461S), SAPK-JNK (CELL9252S), phospho-SAPK-JNK (Thr183/185; CELL9251S) and G6Pase (CELL11712S) from Cell Signaling Technology (Beverly, MA, USA); IL–6 (AB6672) and TNF-alpha (AB9635) from Abcam (Cambridge, UK); and Foxo1 (NB11056991) from Novus Biologicals (Littleton, CO, USA). After washing with TBS containing 0.1% tween–20, all membranes were incubated for 1 h at 4°C with secondary antibody conjugated with a horseradish peroxidase. The specific immunoreactive bands were detected by chemiluminescence (GE Healthcare, ECL Plus Western Blotting Detection System, RPN2132). Images were acquired by the C-DiGit^TM^ Blot Scanner (LI-COR^R^, Lincoln, Nebraska, USA) and quantified using thesoftware Image Studio for C-DiGit Blot Scanner.

### Glycogen concentration measurements

After 12h of fasting, rodents were anaesthetized with an intraperitoneal (i.p.) injection of 2-2-2 tribromoethanol 2.5% (10–20μL.g^−1^) and liver samples (n = 6) were used for glycogen concentration measurements as described by Dubois et al. [23].

### Statistical analysis

Results are expressed as the mean ± standard error of the mean (SE). According to a Shapiro—Wilk’s *W*-test, the data were normally distributed and homogeneity was confirmed by Levene’s test. Therefore, a repeated-measures analysis of variance (ANOVA) was used to examine the effects of OT protocols on the training volume. For the other parameters, a one-way ANOVA was used to examine the effects of the OT protocols. When repeated measures and/or one-way ANOVA indicated statistical significance, a Bonferroni’s post hoc test was performed. All statistical analyses were two-sided and the significance level was set at p< 0.05. Statistical analyses were performed using STATISTICA 8.0 computer software (StatSoft®, Tulsa, OK).

## Results

### Training volume and performance parameters

**Fig 1:**
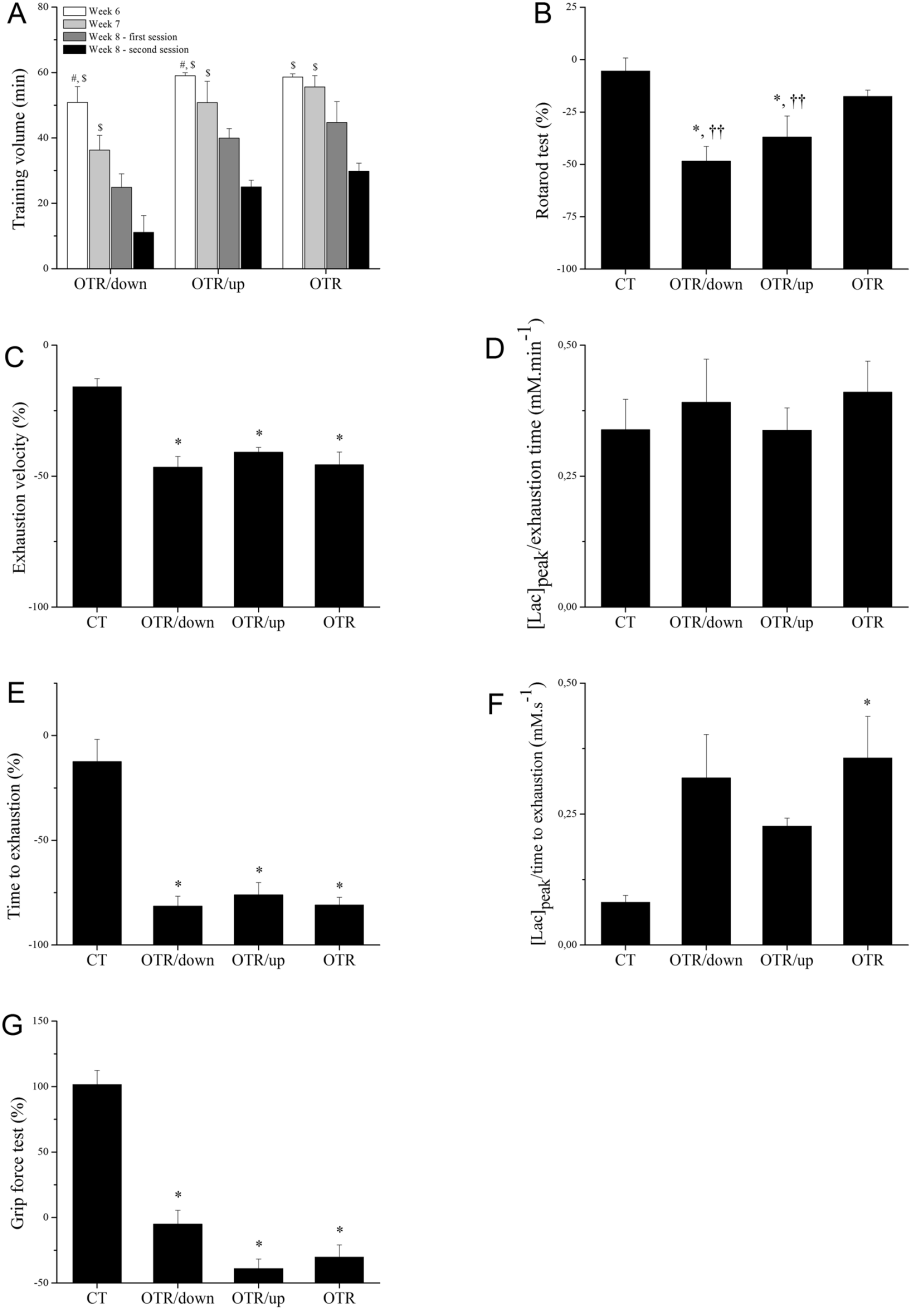
The training volume (min) was measured daily from the fifth week in the OT protocols. Once mice from OTR/down, OTR/up and OTR performed the entire training sessions in the fifth week, the figure presents the data from the sixth to the eighth week (A); Percentage alteration (%) of rotarod test between week 0 and week 8 for the experimental groups (B); Percentage alteration (%) of exhaustion velocity between week 4 and week 8 for the experimental groups (C). Ratio between peak blood lactate concentration ([Lac]_peak_; mM) and exhaustion time (min) measured after the incremental load test at the end of week 8 for the experimental groups (D); Percentage alteration (%) of time to exhaustion between week 0 and week 8 for the experimental groups (E); Ratio between peak blood lactate concentration ([Lac]_peak_; mM) and time to exhaustion (s) measured after the exhaustion test at the end of week 8 for the experimental groups (F); Percentage alteration (%) of grip force test between week 0 and week 8 for the experimental groups (G). Data correspond to means ± SE of n = 12 mice. CT: sedentary mice; OTR/down: overtrained by downhill running; OTR/up: overtrained by uphill running; OTR: overtrained by running without inclination. ^#^P < 0.05 vs.week 8 –first session; ^$^P < 0.05 vs.week 8 –second session; *P < 0.05 vs. CT; ††P < 0.05 vs. OTR.

Fig 1A shows that the training volume (min) measured in week 6 increased over the week 8—first session by 2.1 and 1.5-fold for OTR/down and OTR/up, respectively. Compared with week 8—second session, the training volume of week 6 was 4.6, 2.4 and 2.0-fold higher for OTR/down, OTR/up and OTR, respectively. In addition, the training volume of week 7 was higher than week 8—second session by 3.3, 2.0 and 1.9-fold for OTR/down, OTR/up and OTR, respectively. Fig 1B shows that from week 0 to week 8, the rotarod alteration (%) was higher for OTR/down (-48.5±7.0) and OTR/up (-37.0±10.1) compared with CT (-5.4±6.2) and OTR (-17.6±3.0).

The exhaustion velocity from week 4 to week 8 (Fig 1C) and the time to exhaustion from week 0 to week 8 (Fig 1E) were higher for OTR/down (-46.6±4.1 and -81.4±4.7, respectively), OTR/up (-40.9±1.9 and -76.1±5.9, respectively) and OTR (-45.7±4.9 and -80.9±3.7, respectively) compared with CT (-15.9±3.2 and -12.5±10.6, respectively). Although the [Lac]_peak_/ exhaustion time (mM.min^−1^) that was measured after the ILT at the end of week 8 did not change among the experimental groups (Fig 1D), the [Lac]_peak_/time to exhaustion (mM.s^−1^) that was measured after the exhaustion test at the end of week 8 was higher for OTR (3.5±0.8 × 10^−1^) than for CT (0.8±0.1 × 10^−1^) (Fig 1F). Fig 1G shows that from week 0 to week 8, grip force was lower for OTR/down (-5.0±10.4), OTR/up (-39.0±7.3) and OTR (-30.3±9.3) compared with CT (101.6±10.8).

### Metabolic parameters, GTT, ITT and PTT

**Fig 2:**
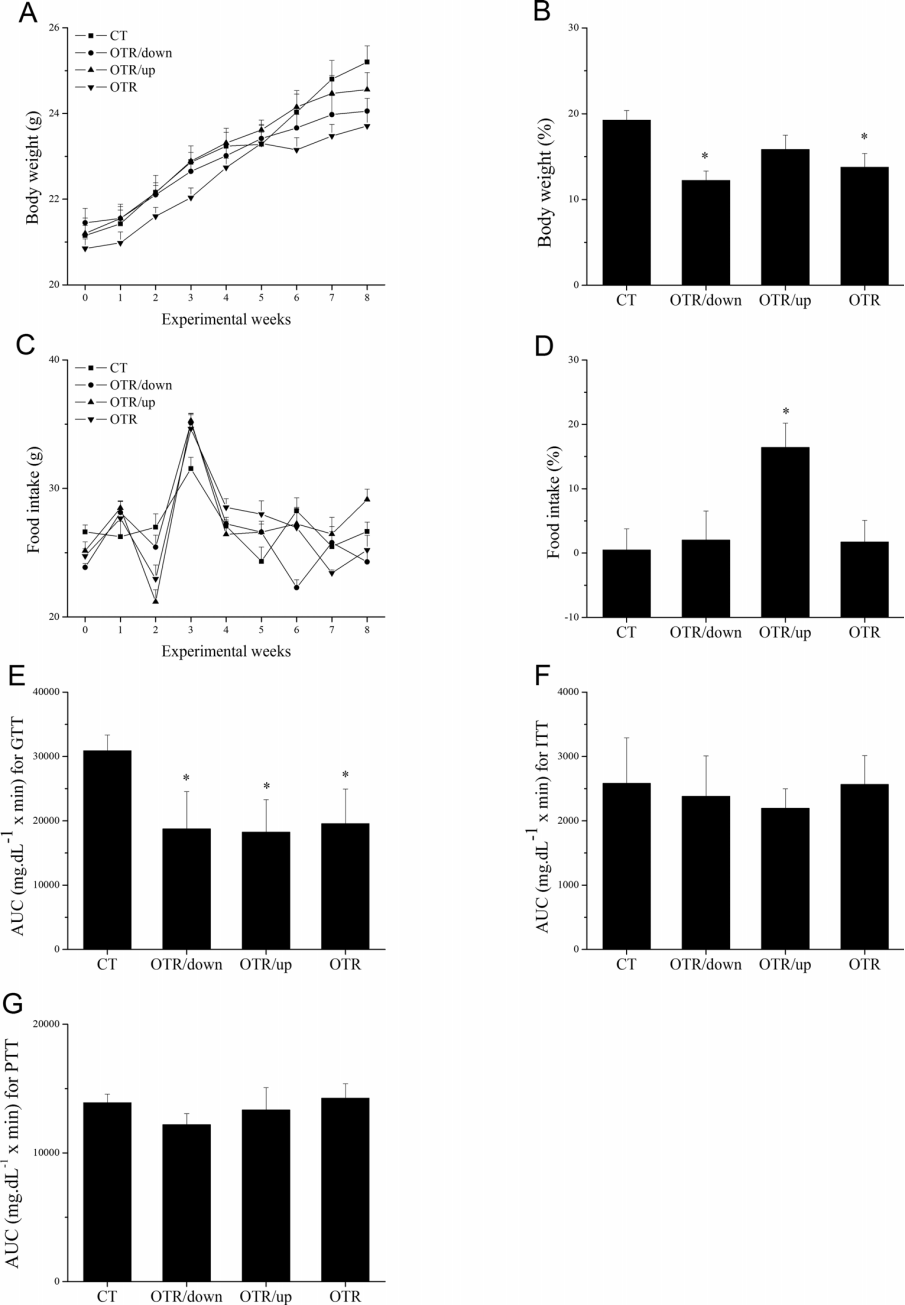
Body weight (g) responses during the experimental weeks for the experimental groups (A); Percentage alteration (%) of body weight between week 0 and week 8 for the experimental groups (B); Food intake (g) responses during the experimental weeks for the experimental groups (C); Percentage alteration (%) of food intake between week 0 and week 8 for the experimental groups (D); The area under curve (AUC; mg.dL^−1^ × min) for the blood glucose responses during 120min after an intraperitoneal injection of glucose (2g.kg^−1^) (E); The area under curve (AUC; mg.dL^−1^ × min) for the blood glucose responses during 30min after an intraperitoneal injection of insulin (1.5 U.kg^−1^) (F). The area under curve (AUC; mg.dL^−1^ × min) for the blood glucose responses during 120min after an intraperitoneal injection of pyruvate (2g.kg^−1^) (G). Data correspond to means ± SE of n = 12 mice. CT: sedentary mice; OTR/down: overtrained by downhill running; OTR/up: overtrained by uphill running; OTR: overtrained by running without inclination. GTT: glucose tolerance test; ITT: insulin tolerance test. PTT: pyruvate tolerance test. *P < 0.05 vs. CT.

Fig 2A and 2C show changes in body weight (g) and food intake (g) for CT, OTR/down, OTR/up and OTR during the experimental weeks. Body weight from week 0 to week 8 was lower for OTR/down (12.2±1.1) and OTR (13.8±1.6) compared with CT (19.3±1.1) (Fig 2B). Fig 2D shows that food intake from week 0 to week 8 was higher for OTR/up (16.4±3.8) than for CT (0.5±3.3). The AUC for the GTT (mg.dL^−1^× min) was lower for OTR/down (18755.0±5792.2), OTR/up (18251.3±5025.1) and OTR (19574.1±5392.2) compared with CT (30892.5±2438.4) (Fig 2E). Fig 2F and 2G show that the AUC for the ITT and PTT (mg.dL^−1^× min) was not different between the experimental groups.

### Insulin, inflammatory and gluconeogenesis signaling pathways

**Fig 3:**
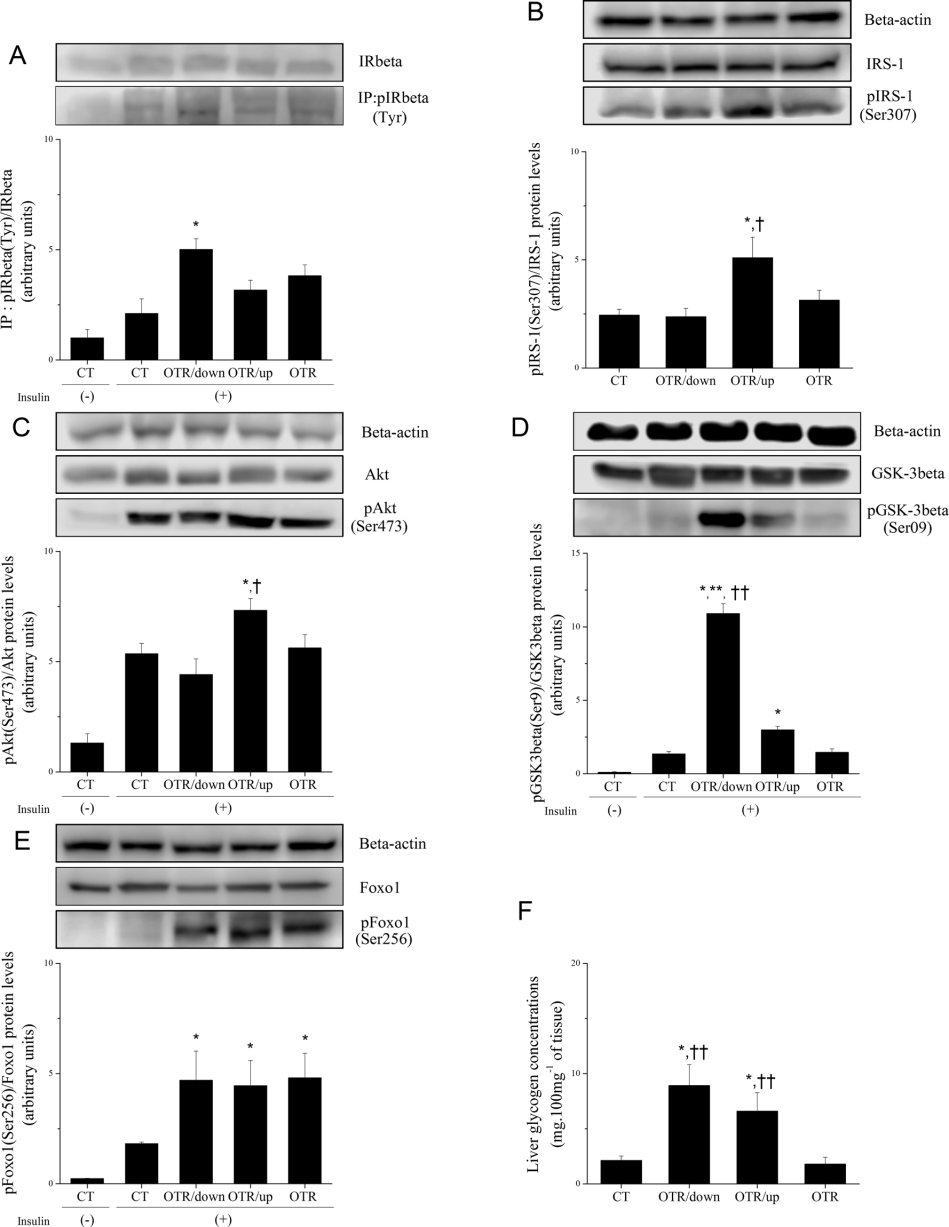
Ratio between pIRbeta (Tyr)/IRbeta (arbitrary units) (A), ratio between pIRS–1 (Ser307)/pIRS–1 (arbitrary units) (B), ratio between pAkt (Ser473)/Akt (arbitrary units) (C), ratio between pGSK3beta (Ser9)/GSK3beta (arbitrary units) (D), and ratio between pFoxo1 (Ser256)/Foxo1 (arbitrary units) (E), and liver glycogen concentrations (mg.100mg^−1^of tissue) (F) for the experimental groups. The blot for IRbeta was performed on total protein liver extracts. Data correspond to means ± SE of n = 6 mice. CT: sedentary mice; OTR/down: overtrained by downhill running; OTR/up: overtrained by uphill running; OTR: overtrained by running without inclination.*P < 0.05 vs. CT. †P < 0.05 vs. OTR/down. **P < 0.05) vs. OTR/up. ††P < 0.05 vs. OTR.

Fig 3A shows that pIRbeta levels for OTR/down increased by 2.4-fold over CT after insulin injection. pIRS–1 levels decreased 2.1(CT) and 2.2-fold (OTR/down) compared with OTR/up (Fig 3B). Fig 3C shows that pAkt levels decreased 1.4 (CT) and 1.7-fold (OTR/down) compared with OTR/up after insulin injection. After insulin injection, pGSK3beta levels increased 8.4 (OTR/down) and 2.2-fold (OTR/up) over CT. In addition, pGSK3beta decreased 3.8 (OTR/up) and 7.3-fold (OTR) compared with OTR/down (Fig 3D). Fig 3E shows that pFoxo1 levels increased 2.6 (OTR/down), 2.5 (OTR/up) and 2.7-fold (OTR) over CT after insulin injection. The liver glycogen concentrations (mg.100mg^−1^ of tissue) of OTR/down (8.9±1.9) and OTR/up (6.9±1.7) were higher than that of CT (2.1±0.4) and OTR (1.8±0.6) (Fig 3F).

**Fig 4:**
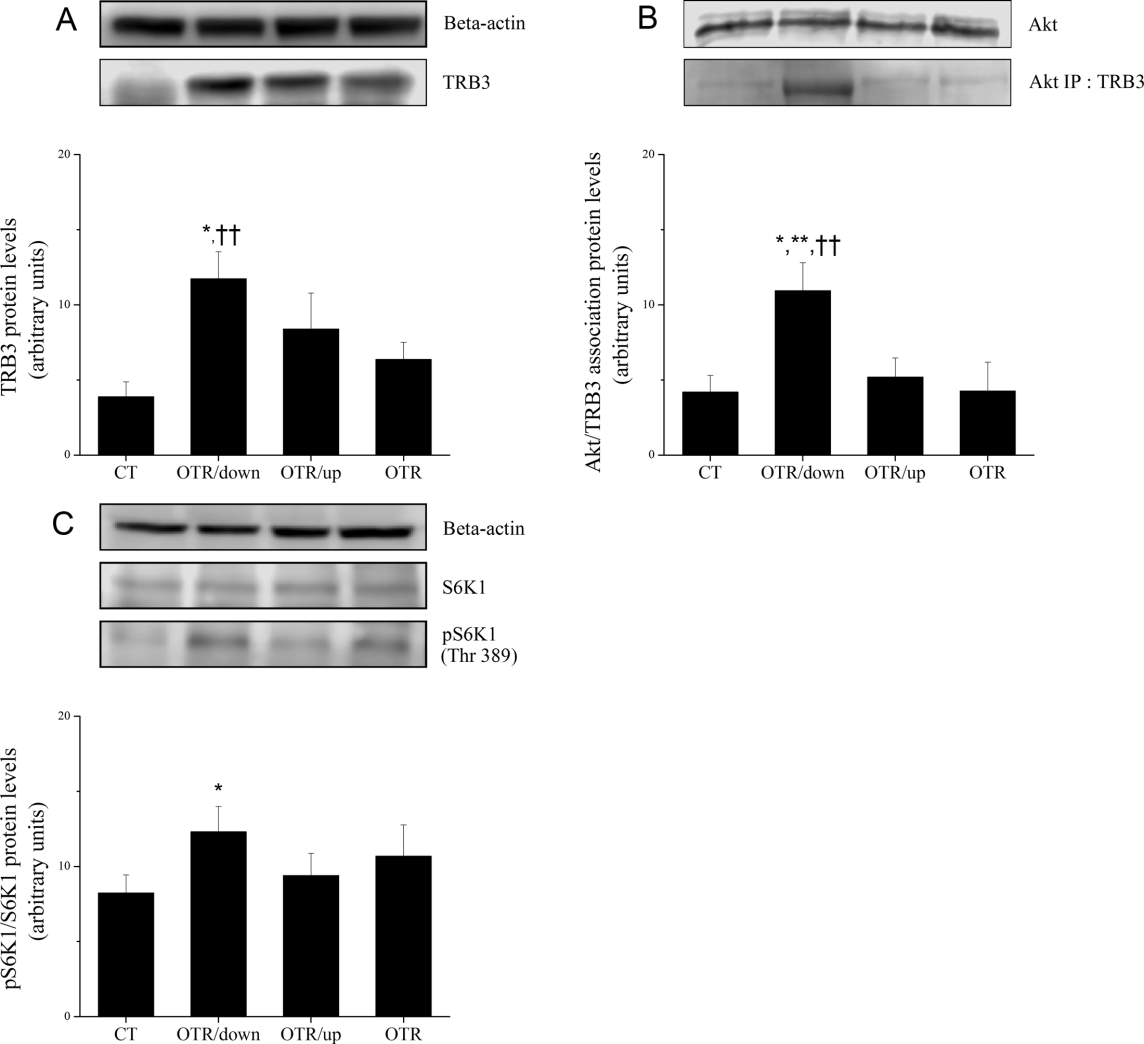
Protein levels (arbitrary units) of TRB3 (A), Akt/TRB3 association (arbitrary units) (B), and ratio between pS6K1 (Thr389)/S6K1 (arbitrary units) (C) for the experimental groups. Data correspond to means ± SE of n = 6 mice. CT: sedentary mice; OTR/down: overtrained by downhill running; OTR/up: overtrained by uphill running; OTR: overtrained by running without inclination.*P < 0.05 vs. CT. **P < 0.05) vs. OTR/up. ††P < 0.05 vs. OTR.

**Fig 5:**
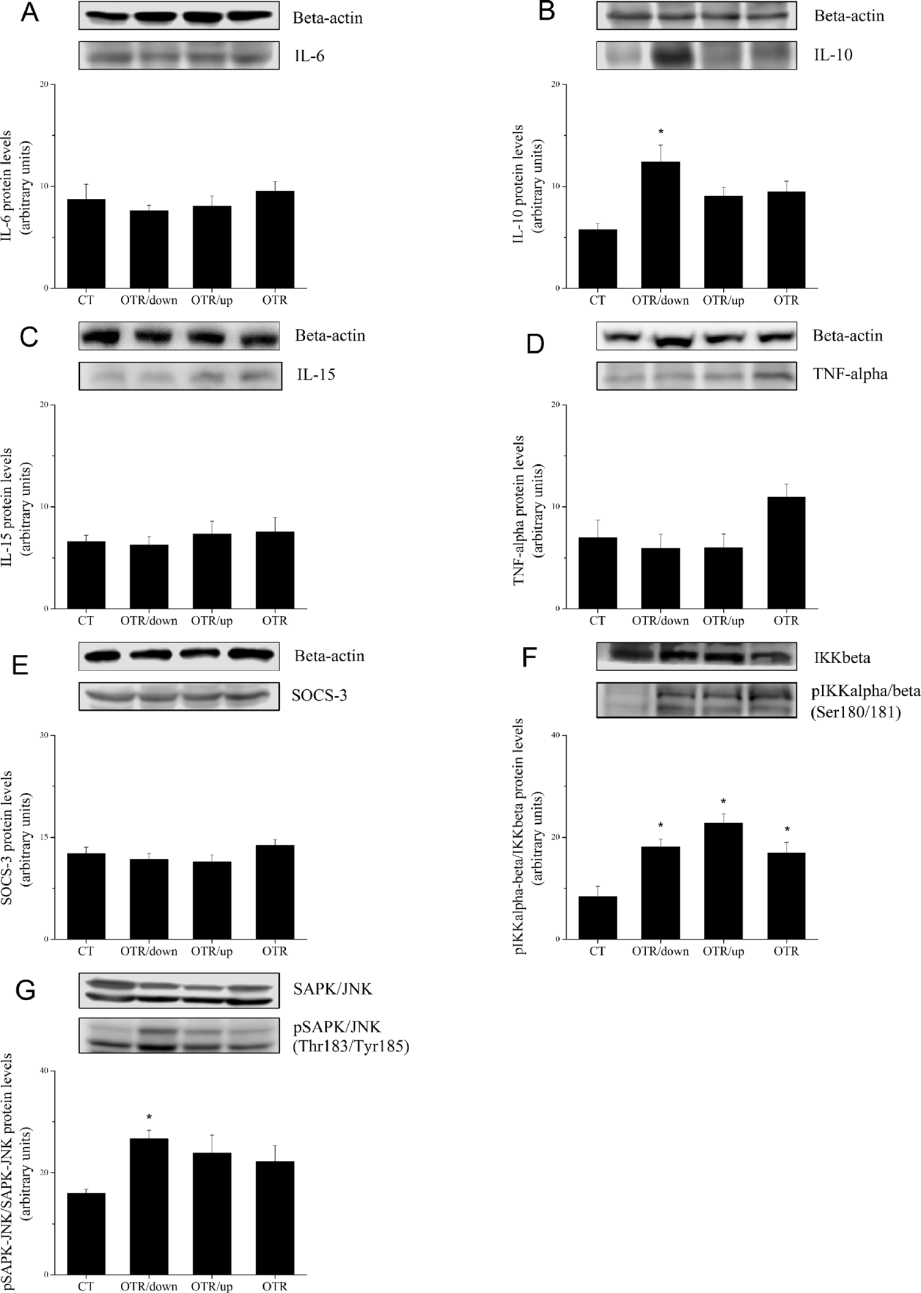
Protein levels (arbitrary units) of IL–6 (A), IL–10 (B), IL–15 (C), TNF-alpha (D), SOCS3 (E) and their respective β-actin controls for the experimental groups. Ratio between pIKKalpha/beta (Ser180/181)/IKKbeta (arbitrary units) (F), and ratio between pSAPK-JNK (Thr183/Tyr185)/SAPK-JNK (arbitrary units) (G) for the experimental groups. Data correspond to means ± SE of n = 6 mice. CT: sedentary mice; OTR/down: overtrained by downhill running; OTR/up: overtrained by uphill running; OTR: overtrained by running without inclination.*P < 0.05 vs. CT.

**Fig 6:**
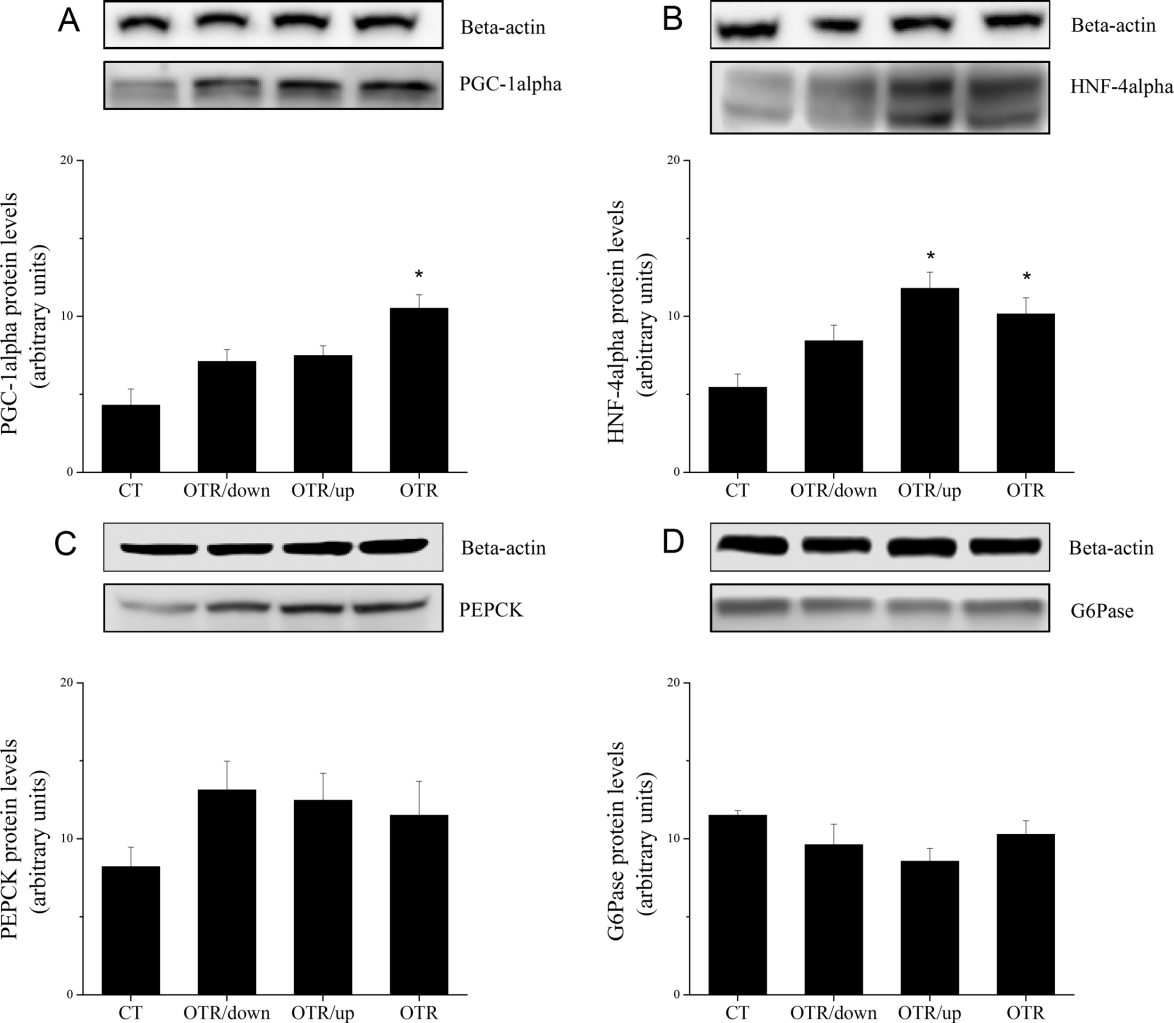
Protein levels (arbitrary units) of PGC-1alpha (A), HNF-4alpha (B), PEPCK (C), G6Pase (D) and their respective β-actin controls for the experimental groups. Data correspond to means ± SE of n = 6 mice. CT: sedentary mice; OTR/down: overtrained by downhill running; OTR/up: overtrained by uphill running; OTR: overtrained by running without inclination. *P < 0.05 vs. CT.

Fig 4A shows that TRB3 protein levels decreased 3.0 (CT) and 1.8-fold (OTR) compared with OTR/down. The association of TRB3 with Akt decreased 2.6 (CT), 2.1 (OTR/up) and 2.6-fold(OTR) compared with OTR/down (Fig 4B). Fig 4C shows that pS6K1 levels of OTR/down increased 1.5-fold over CT. The protein levels of IL–6, IL–15, TNF-alpha and SOCS3 were not different among the experimental groups (Fig 5A and 5C–5E). IL–10 protein levels of OTR/down increased 2.2-fold over CT (Fig 5B). Fig 5F shows that pIKKalfa/beta levels increased 2.2 (OTR/down), 2.7 (OTR/up) and 2.0-fold (OTR) over CT. The pSAPK-JNK levels of OTR/down increased 1.7-fold over CT (Fig 5G). Regarding the gluconeogenic proteins, Fig 6A shows that the PGC-1alpha levels of OTR increased 2.4-fold over CT. HNF-4alpha levels increased 2.2 (OTR/up) and 1.9-fold (OTR) over CT (Fig 6B). The protein levels of PEPCK and G6Pase were not different among the experimental groups (Fig 6C and 6D).

## Discussion

The present investigation tested the effects of OT on insulin, inflammatory and gluconeogenesis signaling pathways in mouse livers. First, we found that OT protocols led to similar responses of exhaustion velocity, time to exhaustion, grip force, and AUC for GTT, ITT and PTT. Second, although the OTR/down protocol improved major proteins involved in hepatic insulin signaling, OTR/up and OTR did not elicit the same responses among all of these proteins. Next, with the exception of pSAPK/JNK and IL–10, several inflammatory proteins respond similarly to the different OT protocols. Finally, although the OTR/down protocol did not alter the content of gluconeogenic mediators, OTR/up and OTR did not elicit the same responses among these proteins. Taken together, our results show that the OTR/down protocol improved hepatic insulin signal transduction without a concomitant decrease in inflammatory proteins. However, the other two OT protocols had varied effects on hepatic insulin, inflammatory and gluconeogenesis pathways.

The current OT protocols did not lead to different responses of most performance parameters, reinforcing the idea that NFOR occurs due to an imbalance between training and recovery and is not influenced by the predominance of the muscle contraction type used during the OT sessions. The effects of the three OT protocols on training volume, exhaustion velocity and time to exhaustion were in line with the recent published results by Pereira et al. [15]. Carmichael et al. [24] compared two acute sessions of downhill or uphill running performed at the same absolute intensity during a 150min training session. They observed that downhill running decreased the voluntary wheel-running recovery and the time to exhaustion compared with uphill running. In the current study, the lack of differences between the OT protocols for most of the performance parameters may be related to the fact that each experimental group was evaluated with regard to the specific treadmill inclination of each OT protocol, allowing the use of the same relative intensity for the studied groups.

Regarding the grip force test, the OT protocols also presented a similar decrease from week 0 to week 8. Interestingly, during the same period, the CT group presented an increase of this force parameter (i.e. 101.6±10.8%). Because this increase was not observed for the forepaws (data not shown), it is possible that mice having to stand up and sustain their body weight on the hindpaws to eat the food located at the cage grill may have influenced the grip force for the hindlimbs. According to Caston et al. [25], the rotarod test evaluates cerebellar deficits in rodents and can be influenced by cardiorespiratory endurance, motor coordination and learning. We verified that OTR/down and OTR/up diminished their performance from week 0 to week 8.

As the exhaustion velocity results (a cardiorespiratory endurance parameter) were similar between OTR/down, OTR/up and OTR, it is possible that the differences in the rotarod test results occurred due to motor coordination or learning. In fact, Huang et al. [26] showed that eight-week treadmill training at 70% of the maximal oxygen uptake increased rotarod test performance and was related to the increase of the dendritic density of Purkinje cells (i.e. motor coordination). Considering that the rotarod test and OTR protocol were performed ata horizontal plane, learning may have influenced the results of the rotarod test in the OT protocol performed without inclination.

The low percentage alterations from week 0 to week 8 for body weight and food intake observed in OTR/down and OTR are in accordance with the recent data published by Pereira et al. [15]. This finding reinforces the fact that the observed decreases in body weight may have resulted from hypermetabolism and proteolysis in response to high training loads and insufficient recovery periods [14]. As previously observed [15], the lack of body weight decrease in the OTR/up may be explained by the significant increase of food intake that is likely associated with the high energetic demand of uphill running [27, 28].

The current OT protocols improved the AUC for the GTT over CT, but these protocols did not influence the AUC for the ITT. Recently, Pereira et al. [5] observed that muscle insulin signaling is impaired after OTR/down but lacks a significant differences in the ITT. They hypothesized that other tissues, including the liver, may not exhibit an impairment in insulin signaling and could play an important role in the maintenance of glucose homeostasis. In the current study, our previous hypothesis that liver mediates glucose homeostasis during overtraining [5] was confirmed and supported other investigations showing an improvement in glucose tolerance in mice that were unable to synthesize muscle glycogen [29, 30]. In fact, we verified that some of the proteins related to hepatic insulin signaling (i.e. pIRbeta, pGSK3beta and pFoxo1) were improved by the OTR/down protocol.

Because TRB3 is a suppressor of hepatic Akt activity [9, 31], we consider that the elevated levels of TRB3 and its association with Akt for OTR/down were responsible by the lack of difference in pAkt levels, even with high levels of pIRbeta. Insulin-mediated Akt activation leads to phosphorylation and inhibition of GSK3beta, activating glycogen synthase and increasing hepatic glycogen deposition [9]. Here, OTR/down caused high levels of pGSK3beta with a concomitant increase in liver glycogen concentrations. Because the serum-glucocorticoid regulated kinase 1 (SGK1) in liver with specific deletion of Akt1 and Akt2 [32], and the ribosomal protein S6 kinase 1 (S6K1) in liver with Akt attenuation [33] are able to phosphorylate and inhibit GSK3beta, it is possible that the high levels of pS6K1observed in the OTR/down was important in modulating pGSK3beta levels even without significant alterations in pAkt levels.

Compared with OTR/down, OTR/up and OTR did not impact hepatic insulin signaling mediators in a similar manner. Although pIRS–1(Ser307) upregulation was directly related to muscle insulin signaling impairment after OTR/down [5], here we verified that OTR/up caused a concomitant increase in pIRS–1 (Ser307) and pAkt levels. The role of pIRS–1 (Ser307) in the control of insulin signaling is undisputed; however, there is little consensus on whether pIRS–1 is part of positive [34] or negative [35] feedback. Recently, Rajanand coworkers [36] highlighted the importance of the cell type and experimental conditions in attributing a positive or negative role for pIRS–1 (Ser307) in the insulin pathway. Even with high levels of pAkt, OTR/up had lower pGSK3beta compared to OTR/down, reinforcing the possibility that other molecules independent of Akt may be phosphorylating and inhibiting pGSK3beta in response to OTR/down and OTR/up. As observed for OTR/down, OTR/up also elevated liver glycogen concentrations.

In addition to GSK3beta inhibition, insulin-mediated Akt activation leads to Foxo1 phosphorylation and blocks the expression of a number of genes involved in gluconeogenesis such as PEPCK and G6Pase [9, 37]. Here, OTR/down and OTR caused high levels of pFoxo1 without significant alterations of pAkt. Because ~84% of liver Akt exists in the Akt2 isoform [32], and we measured the levels of Akt1 phosphorylation (Ser473), it is possible that the high levels of pFoxo1 in OTR/down and OTR occurred due to Akt2 phosphorylation (Ser474). Although Lee et al. [38] showed that Foxo1 (Ser256) may also be phosphorylated by the cAMP-dependent protein kinase (PKA) in vascular endothelial cells, the effects of PKA and other AGC family kinases on pFoxo1 in different experimental conditions and tissues remain to be determined.

The elevated levels of pIKKalpha/beta and pSAPK/JNK in the livers of OTR/down compared to CT are in accordance with our previously published results analyzing skeletal muscle samples [5]. However, different from the muscle samples [5], the high levels of these proteins were not associated with high levels of pIRS–1 (Ser307). While the OTR/up and OTR presented the same results of OTR/down for pIKKalpha/beta, they did not differ from CT for pSAPK/JNK. Although high levels of pIKKalpha/beta and pSAPK/JNK are associated with high levels of pIRS–1 (Ser307) in non-alcoholic fatty liver disease [39], it is unclear whether this relationship occurs in the livers of NFOR mice. Because the anti-inflammatory cytokine IL–10 protects against insulin resistance by inhibiting the action of IKK on pIRS–1 (Ser307) [40], we consider the high levels of IL–10 found in OTR/down were important in maintaining pIRS–1 (Ser 307) levels even with high levels of pIKKalpha/beta.

Hepatic PGC1-alpha binds to and coactivates transcription factors such as HNF-4alfa and Foxo1, coordinating the expression of rate-limiting gluconeogenic genes (i.e. PEPCK and G6Pase) during insulin resistance [9, 37]. Here, we observed high levels of PGC-1alpha in OTR and of HNF-4alpha in OTR/up and OTR without significant alterations in PEPCK and G6pase levels, which accounts for the lack of differences for PTT among the groups. These results may be explained by two hypotheses. First, the lack of PEPCK and G6pase suppression even with high levels of pFoxo1 in OTR/down, OTR/up and OTR is in line with Lu´s investigation [32] showing that the expression of these gluconeogenic genes was not altered in livers with the acute deletion of Akt1, Akt2 and Foxo1, during fasting or after feeding. The second hypothesis is based on the conclusion of Haase et al. [41] that PGC-1alfa is not required for the increase in liver PEPCK levels after chronic exercise or for the increase in liver G6Pase mRNA levels after acute exercise.

## Conclusions

In summary, the EE sessions-induced OT protocol improved the major proteins of the hepatic insulin signaling pathway in mouse livers but up-regulated TRB3 and its association with Akt. The other OT protocols performed in uphill and without inclination did not lead to the same responses for all of the proteins related to hepatic insulin, inflammatory and gluconeogenesis pathways. These results indicate that liver acts as a compensatory organ when skeletal muscle presents insulin signaling impairment, suggesting that the liver must be investigated more thoroughly in NFOR states.

## Supporting Information

S1 ARRIVE Checklist NC3Rs ARRIVE Guidelines Checklist 2014. (DOCX)

S1 File Mean ± SE of the experimental groups for Figs 1–6. (ZIP)
